# Lower Ipsilateral Hippocampal Integrity after Ischemic Stroke in Young Adults: A Long-Term Follow-Up Study

**DOI:** 10.1371/journal.pone.0139772

**Published:** 2015-10-13

**Authors:** Pauline Schaapsmeerders, Anil M. Tuladhar, Noortje A. M. Maaijwee, Loes C. A. Rutten-Jacobs, Renate M. Arntz, Hennie C. Schoonderwaldt, Lucille D. A. Dorresteijn, Ewoud J. van Dijk, Roy P. C. Kessels, Frank-Erik de Leeuw

**Affiliations:** 1 Radboud University Medical Centre, Donders Institute for Brain, Cognition and Behaviour, Department of Neurology, Nijmegen, the Netherlands; 2 Department of Clinical Neurosciences, University of Cambridge, Cambridge, United Kingdom; 3 Department of Neurology, Medisch Spectrum Twente, Enschede, the Netherlands; 4 Donders Institute for Brain, Cognition and Behaviour, Centre for Neuroscience and Centre for Cognition, Radboud University Nijmegen, Nijmegen, the Netherlands; 5 Department of Medical Psychology, Radboud university medical centre, Nijmegen, the Netherlands; University of Pécs Medical School, HUNGARY

## Abstract

**Background and purpose:**

Memory impairment after stroke is poorly understood as stroke rarely occurs in the hippocampus. Previous studies have observed smaller ipsilateral hippocampal volumes after stroke compared with controls. Possibly, these findings on macroscopic level are not the first occurrence of structural damage and are preceded by microscopic changes that may already be associated with a worse memory function. We therefore examined the relationship between hippocampal integrity, volume, and memory performance long after first-ever ischemic stroke in young adults.

**Methods:**

We included all consecutive first-ever ischemic stroke patients, without hippocampal strokes or recurrent stroke/TIA, aged 18–50 years, admitted to our academic hospital between 1980 and 2010. One hundred and forty-six patients underwent T1 MPRAGE, DTI scanning and completed the Rey Auditory Verbal Learning Test and were compared with 84 stroke-free controls. After manual correction of hippocampal automatic segmentation, we calculated mean hippocampal fractional anisotropy (FA) and diffusivity (MD).

**Results:**

On average 10 years after ischemic stroke, lesion volume was associated with lower ipsilateral hippocampal integrity (p<0.05), independent of hippocampal volume. In patients with a normal ipsilateral hippocampal volume (volume is less than or equal to 1.5 SD below the mean volume of controls) significant differences in ipsilateral hippocampal MD were observed (p<0.0001). However, patients with a normal hippocampal volume and high hippocampal MD did not show a worse memory performance compared with patients with a normal volume and low hippocampal MD (p>0.05).

**Conclusions:**

Patients with average ipsilateral hippocampal volume could already have lower ipsilateral hippocampal integrity, although at present with no attendant worse memory performance compared with patients with high hippocampal integrity. Longitudinal studies are needed to investigate whether a low hippocampal integrity after stroke might lead to exacerbated memory decline with increasing age.

## Introduction

Although episodic memory dysfunction frequently occurs after ischemic stroke at young age (18 through 50 years), it's underlying mechanism is poorly understood. That is, stroke typically does not affect brain structures that are crucial for memory formation and retrieval, such as the hippocampus [1]. However, recent studies started to unravel potential underlying mechanisms by demonstrating smaller *ipsilateral* hippocampal volumes in patients who experienced an ischemic stroke outside the medial temporal lobe at young age, with an accompanying worse memory performance [2].

Most likely, these findings on the macroscopic level are not the first manifestation of structural damage of the hippocampus after ischemic stroke as they are presumably preceded by microstructural changes with already (subtle) cognitive correlates. Diffusion Tensor Imaging (DTI) is able to detect these early manifestations of damage on the microstructural level [3]. Therefore, in young stroke patients these microstructural changes may explain lower verbal memory performance, even before structural changes on conventional MRI appear. However, to the best of our knowledge, this has never been investigated in patients with stroke at young age, whereas understanding of the pathophysiology of post-stroke memory dysfunction is of particular importance in young patients, since they are in a period of life in which they start forming a family, have an active social life, and make decisive career moves, requiring optimal cognitive function.

Therefore, we investigated long-term hippocampal integrity in patients with first-ever young ischemic stroke. This study is embedded within the “*F*ollow-*U*p of *T*ransient ischemic attack and stroke patients and *U*nelucidated *R*isk factor *E*valuation”-study (*FUTURE* study) that addresses risk factors and prognosis after stroke in young adults.

## Patients and Methods

### Study design

This study is part of the *FUTURE* study, a prospective cohort study of prognosis after transient ischemic attack (TIA), ischemic stroke, or hemorrhagic stroke in adults aged 18 through 50 years admitted to the Radboud university medical centre, the Netherlands, between January 1, 1980, and November 1, 2010 [4–6]. The Medical Review Ethics Committee region Arnhem-Nijmegen approved the study and written informed consent was obtained from all participants.

Patients were identified through a prospective registry of all consecutive young stroke/TIA patients that has been kept at the department since the 1970s with a standardized collection of baseline and clinical characteristics (including demographics, stroke subtype, vascular risk factors, and a history of epilepsy). Ischemic stroke was defined as focal neurologic deficit persisting more than 24 hours. Lesion location (left hemisphere, right hemisphere, and infratentorial) was based on medical records and radiological findings. Lesion location was further specified into frontal lobe, parietal lobe, temporal lobe, occipital lobe, basal ganglia, thalamus, brainstem, and cerebellum based on the T1-weighted images and FLAIR sequence. Patients had the opportunity to undergo an extensive neuropsychological examination after long follow-up which was administered between November 2009 and December 2011 [6].

The present substudy comprises all consecutive patients with a first-ever ischemic stroke. Primary exclusion criteria for ischemic stroke in the FUTURE study were previous stroke or TIA, cerebral venous sinus thrombosis, and retinal infarction [5]. Additional exclusion criterion for the present substudy were recurrent stroke/TIA and hippocampal stroke.

Stroke-free control participants were recruited among the patients’ spouses, relatives, or social environment. Inclusion criteria were: 18 years or older without a history of TIA or stroke. The control group and patient group were matched for age, sex, and level of education. Controls were all living independently, none fulfilling the clinical criteria of dementia.

### Episodic memory

Episodic memory performance was evaluated with the Rey Auditory Verbal Learning Test [RAVLT][7], a widely used word-list learning test tapping verbal memory function. The RAVLT consists of three consecutive learning trails, followed by a delayed free recall test as well as a delayed recognition test. In the present study we used the immediate verbal recall score (total number of correctly recalled words over the three consecutive learning trials) and the delayed verbal recall score since these measures are most sensitive to hippocampal (dys)function and previous studies found a clear association with hippocampal integrity [8–12]. Immediate verbal recall reflects the encoding phase of memory functioning. Delayed verbal recall after the learning trials of the RAVLT reflects storage and retrieval success of previously acquired information [13].

### Other measurements

Level of education was scored with a widely-used Dutch scoring system (1 = less than primary school; 7 = university degree). Functional outcome during follow-up visit was evaluated using the modified Rankin Scale (mRS) [14]. Furthermore, assessment of stroke etiology (TOAST)[15] and severity (National Institutes of Health Stroke Scale, NIHSS)[16] was done retrospectively for all cases by a validated approach [17, 18], as these scales did not exist at the time when a substantial proportion of the patients experienced their qualifying event. The etiology of stroke using TOAST was classified as atherothrombotic stroke, cardioembolic stroke, lacunar stroke, rare causes (vasculitis, moyamoya, migraine, coagulopathy, systemic disease, genetic, toxic, post-surgery, traumatic, pregnancy related), multiple causes, or unknown cause. Depressive symptoms and fatigue were assessed using the depression subscale of the Hospital Anxiety and Depression Scale (HADS)[19] and the subscale Subjective Fatigue of the revised Checklist Individual Strength (CIS-20R) [20].

We assessed vascular risk factors at follow-up (hypertension, diabetes mellitus, dyslipidemia, smoking (current/former/never), current alcohol use (>2 units/day)) on the basis of medical history using a standardized, structured questionnaire and/or the use of medication. The Body-mass index (BMI) at follow-up was calculated as weight (kilograms) divided by height (meters) squared.

### Neuroimaging data acquisition

Participants underwent a 1.5-T MRI scanning on the Siemens, Magnetom Avanto. A T1-weighted whole-brain scan was collected (magnetization-prepared rapid acquisition with gradient echo [MPRAGE], TI 1000 ms, repetition time [TR] 2730 ms, echo time [TE] 2.95 ms, flip angle 7°, field of view [FOV] = 256 mm, voxel size 1.0×1.0×1.0 mm^3^) as well as a set of whole-brain diffusion-weighted images (TR 9100 ms, TE 98 ms, diffusion directions 61, with non co-linear orientation of the diffusion-weighting gradient and b-value 1000 s/mm^2^. Seven unweighted images, FOV = 220 mm, voxel size 2.2×2.2×2.2 mm^3^) and a FLAIR pulse sequence (TI /TR/TE 2200 ms/12220 ms/85 ms; interslice gap: 0.6 mm; voxel size 1.2x1.0x3.0 mm^3^).

### Neuroimaging data processing

#### Hippocampal and intracranial volumetry

Neuroimaging data analyses are extensively described elsewhere [2]. In short, neuroimaging data were analyzed using Matlab 7. Voxel-Based Morphometry toolbox (VBM8) within SPM8 was used for each T1-weighted image to determine the volume of grey matter, white matter, and CSF to calculate the intracranial volume (ICV).

Hippocampal volumes were determined by automatic segmentation (FSL-FIRST: FMRIB Software Library release 5.0, http://www.fmrib.ox.ac.uk/fsl) with manual correction using FSLview by one experienced investigator (PS) who was blinded for baseline characteristics and memory performance. The anatomical boundaries were determined using a previously published standardized protocol in which segmentation correction was performed from posterior to anterior [7, 21]. Normalized brain volume and hippocampal volume (ml) were calculated with the following formula: (average intracranial volume of the total population * (brain or hippocampal volume of the participant/intracranial volume of the participant)) [22]. Inter-rater reliability on 10% of all cases showed an intra-class correlation coefficient for the left hippocampus of 0.81, and for the right hippocampus of 0.83. The intra-rater reliability for hippocampal volumes yielded an intra class correlation coefficient for the left hippocampus of 0.93 and for the right hippocampus 0.96.

#### Thalamus

To determine whether the results are specific for the hippocampus or not, we additionally investigated thalamic integrity. Lesion studies have shown that, apart from the hippocampus, thalamic lesions have also been found to be associated with memory dysfunction [23] and previous studies have shown that ipsilateral thalamic volume is reduced after ischemic stroke [2, 24, 25]. Thalamic volume was determined using FSL-FIRST [26]. Patients with a thalamic stroke were excluded from the analysis. Normalized thalamic volumes were calculated with the same formula used to normalize hippocampal volumes.

#### DTI analysis

First, diffusion data were preprocessed to detect and correct head and cardiac motion artifacts, using an in-house developed iteratively re-weighted-least-squares algorithm named ‘PATCH’ [27]. Eddy current corrections and motion artifacts from affine misalignment are performed simultaneously by minimization of the residual diffusion tensor errors. Using DTIFit within the Functional MRI of the Brain Diffusion Toolbox, we created FA en MD images. Next, the mean unweighted image (b = 0) was used to coregister to the anatomical T1 image using SPM8 with default settings, which were then applied to the FA en MD images. All images were visually inspected for severe motion artefacts and co-registration errors. To reduce partial volume effects for hippocampal and thalamic MD and FA, we eroded the hippocampus and thalamic volumes by one voxel in all directions[8]. All images were checked for not including peri-hippocampal CSF. The mean FA and MD were calculated for the right and left hippocampus and thalamus.

#### Ischemic stroke volume and lesion probability maps

Ischemic lesions were defined as hypointense areas on a T1-weighted MPRAGE whole-brain scan with corresponding gliotic rim on FLAIR. The T1-weighted image from each patient was used to manually trace lesions, with the aid of corresponding slices on the FLAIR image. One experienced investigator traced all the lesions and was blinded for baseline characteristics and outcome measures. Normalized lesion volumes (ml) were calculated with the same formula used to normalize hippocampal volume. Next, the T1-weighted images were brain-extracted (FSL-BET: Brain Extraction Tool)[28] and subsequently, along with the lesion mask, registered to the Montreal Neurological Institute (MNI) standard space by an affine transformation with 12 degrees of freedom using FSL-FLIRT (FMRIB’s Linear Image Registration Tool; Software Library release 5.0, http://www.fmrib.ox.ac.uk/fsl), followed by non-linear registration using FNIRT (FMRIB’s Non-linear Image Registration Tool). Next, for each patient group all lesion masks were merged and averaged, which resulted in a lesion probability map for left-hemispheric stroke, right-hemispheric stroke, and infratentorial stroke patients.

### Statistical analyses

Baseline characteristics were presented as means (±SD), median (Q1-Q3), or number of cases (%). To investigate whether participants in the present DTI study differed from non-participants on basic demographical and clinical characteristics we used a t-test in case of normal distributed data (age at event and follow-up), a Mann-Whitney U test in case of ordinal data (NIHSS at admission), and Pearson’s chi-square test in case of categorical data (sex, TOAST, lesion location). For the participants, group differences between the three groups of patients (left/right/infratentorial stroke) on basic demographical and clinical characteristics were investigated using a t-test, Mann-Whitney U test (two groups), Kruskal-Wallis test (three groups), or Pearson’s chi-square test when appropriate. Since the explanatory nature of this study, two-tailed p-values<0.05 were considered significant.

We investigated whether different lesion locations (left hemisphere/right hemisphere/infratentorial) were associated with differences in left and right hippocampal integrity compared with a non-stroke population. We therefore calculated mean FA and MD for the left and right hippocampus separately, stratified by stroke location (left hemisphere/right hemisphere/infratentorial) and were compared with controls by means of ANCOVA, adjusted for age at follow-up and sex.

We investigated whether lesion volume was associated with lower ipsilateral hippocampal integrity in hemispheric stroke, independent from hippocampal volume. Linear regression was used for this purpose adjusting for, age, sex, follow-up duration, lesion location (left/right), and ipsilateral hippocampal volume. To investigate the relation between ipsilateral hippocampal volume and ipsilateral hippocampal integrity, the same linear regression model was used as to investigate the relation between lesion volume and hippocampal integrity and we subsequently reported whether there was an independent association between ipsilateral hippocampal volume and ipsilateral hippocampal integrity.

Finally, we investigated whether there were patients with normal ipsilateral hippocampal volume and lower ipsilateral hippocampal integrity and possibly already an attendant worse memory performance compared with patients with normal volume and high hippocampal integrity. We used controls as a reference for normal hippocampal volume. To account for the effect of ageing on hippocampal volume[29] we divided the control group into two equal groups based on the median age of the control group. Next, we used the mean hippocampal volume and SD of these two age groups to calculate an age-adjusted Z-score of hippocampal volume for each participant. Hippocampal atrophy was defined as more than 1.5 below the mean volume of controls [30, 31]. Normal hippocampal volume was defined as less than or equal to 1.5 SD below mean volume of controls. Next, we excluded patients with thalamic stroke from the analysis to investigate the independent effect of the hippocampus. For left and right-hemispheric stroke patients separately, patients with normal ipsilateral hippocampal volume were split into two equal groups based on the median of ipsilateral hippocampal MD: low ipsilateral hippocampal MD (lowest 50%) and high ipsilateral hippocampal MD (highest 50%). We investigated whether these two patient groups with normal volume and low versus high hippocampal MD significantly differed in hippocampal MD, using an ANCOVA model, adjusted for age, sex, follow-up duration, and ipsilateral hippocampal volume. Next, we investigated whether hippocampal volume significantly differed between these patients with high or low hippocampal MD, using an ANCOVA model, adjusted for age, sex, and follow-up duration. Last, we investigated in patients with normal volume whether there is a difference in memory performance between patients with low hippocampal MD and high hippocampal MD. In this way, we investigated the additional effect of higher hippocampal MD on memory performance in stroke patients. An ANCOVA model was used for this purpose adjusted for age, sex, education, follow-up duration, and ipsilateral hippocampal volume.

The described analyses on memory performance were also performed for patients with normal thalamic volume and low or high ipsilateral thalamic integrity (thalamic stroke excluded), to investigate whether present results were specific for the hippocampus.

## Results

T1-weighted whole-brain imaging, DTI-scanning (no artefacts), and memory performance was available from 146 ischemic stroke patients and 84 stroke-free controls. Baseline characteristics between participants and those who refused or were lost to follow-up were not significantly different. Those who participated in the FUTURE study, but did not participate in the present study had lower education (p = 0.03) and a higher mRS at follow-up (p = 0.0001) compared with participants who did participate in the present DTI study.

**Fig 1:**
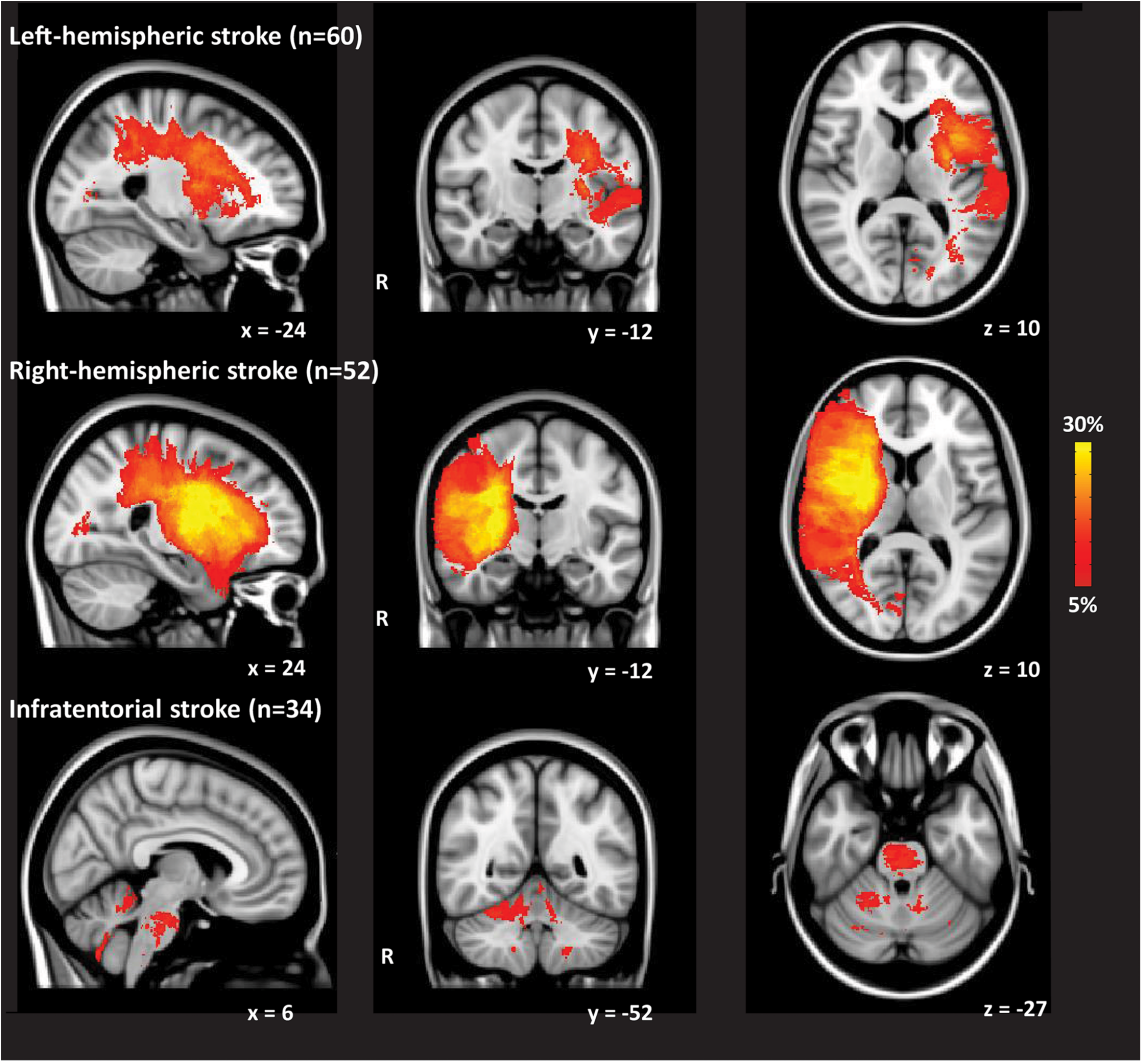
Lesion probability maps in patients with left-hemispheric stroke, right-hemispheric stroke, and infratentorial stroke. The color overlay created on top of the Montreal Neurologic Institute (MNI) standard brain template shows the probability of each voxel containing a lesion in each patient group. The color bar denotes the probability range.

Table 1 shows the baseline characteristics of the study population and group comparisons between the three groups of patients. Mean follow-up duration was 10.4 years (SD 8.0) and mean age at follow-up was 50.0 years (SD 9.8). Mean age of controls was 48.9 years (SD 11.9). Infratentorial stroke patients had a significant higher proportion of men compared with left-hemispheric stroke patients (p = 0.002) and right-hemispheric stroke patients (p = 0.003) (Table 1). Left-hemispheric stroke patients reported more depressive symptoms compared with infratentorial stroke patients (p = 0.02). Based on the NIHSS at admission, right-hemispheric stroke patients had a more severe stroke compared with left-hemispheric stroke patients (p = 0.008). Infratentorial stroke patients had a more severe stroke compared with left-hemispheric stroke patients (p = 0.03), but did not differ in stroke severity compared with right-hemispheric stroke patients (p = 0.6). Right-hemispheric stroke patients had a significant higher mean lesion volume compared with left-hemispheric stroke patients (p = 0.01) and infratentorial stroke patients (p = 0.0004). Whereas, left-hemispheric stroke patients had a significant higher mean lesion volume compared with infratentorial stroke patients (p = 0.02). Left-hemispheric stroke patients and right-hemispheric stroke patients showed the highest lesion probability in the Medial Cerebral Artery (MCA) territory (Fig 1). Infratentorial stroke patients had the highest lesion probability in right cerebellum and pons (Fig 1).

**Table 1:** Demographic and clinical characteristics of ischemic stroke patients and controls.

Characteristics	Total ischemic stroke population	Hemispheric stroke		Infratentorial stroke	Controls	p-value^b^
		Left	Right			
No.	146	60	52	34	84	
Mean age at event (SD)	39.6 (8.2)	39.7 (8.1)	39.0 (8.4)	40.4 (8.1)		0.7
Mean follow-up duration (SD)	10.4 (8.0)	10.6 (8.1)	10.0 (7.9)	10.7 (8.3)		0.9
Mean age at follow-up (SD)	50.0 (9.8)	50.3 (9.4)	49.0 (9.5)	51.2 (11.1)	48.9 (11.9)	0.6
Men, No (%)	67 (45.9%)	21 (35.0%)	23 (44.2%)	23 (67.6%)	38 (45.2%)	0.01
Education, median (Q1-Q3)	5 (4–6)	5 (4–6)	5 (4–5.75)	5 (5–6)	5 (5–6)	0.3
NIHSS at stroke onset, median (Q1-Q3)	4 (2–8)	3 (2–5)	5 (3–10)	5 (2.5–8)		0.01
Normalized total brain volume, ml (SD)	1127.4 (59.4)	1130.8 (61.4)	1107.3 (64.6)	1152.2 (31.9)	1156.6 (32.9)	
Normalized lesion volume, ml (SD)	20.2 (42.0)	15.4 (33.3)	37.7 (56.2)	2.1 (5.1)		0.0002
MRS at follow-up (Q1-Q3)	1 (0–1)	1 (0–1.75)	1 (0–2)	1 (0–1)	0 (0–0)	0.5
HADS—depressive symptoms		4.1 (3.9)	3.2 (3.2)	2.3 (2.8)	2.5 (2.8)	0.04
CIS-20R—subjective fatigue		30.9 (14.3)	27.6 (11.9)	24.2 (15.0)	23.05 (12.7)	0.08
**TOAST, No (%)**						0.5
Atherothrombotic stroke	34 (23.3%)	11 (18.3%)	14 (26.9%)	9 (26.5%)		
Cardioembolic stroke	10 (6.8%)	7 (11.7%)	1 (1.9%)	2 (5.9%)		
Lacunar stroke	17 (11.6%)	10 (16.7%)	4 (7.7%)	3 (8.8%)		
Rare causes	28 (19.2%)	9 (15.0%)	12 (23.1%)	7 (20.6%)		
Multiple causes	2 (1.4%)	0 (0.0%)	1 (1.9%)	1 (2.9%)		
Unknown cause	55 (37.7%)	23 (38.3%)	20 (38.5%)	12 (35.3%)		
**Lesion location, No (%)**						
Left supratentorial stroke	60 (41.1%)					
Right supratentorial stroke	52 (35.6%)					
Infratentorial stroke	34 (23.3%)					
Cortical stroke^a^						
Frontal lobe		35 (58.3%)	33 (63.5%)			
Parietal lobe		24 (40.0%)	26 (50.0%)			
Temporal lobe		21 (35.0%)	28 (53.8%)			
Occipital lobe		14 (23.3%)	8 (15.4%)			
Subcortical stroke^a^						
Basal ganglia		22 (36.7%)	27 (51.9%)			
Thalamus		7 (11.7%)	13 (25.0%)			
Brainstem stroke^a^				24 (70.6%)		
Cerebellar stroke^a^				15 (44.1%)		

Data are expressed as mean (SD), number (%), or median (Q1–Q3).

NIHSS = National Institutes of Health Stroke Scale

mRS = modified Rankin Scale.

HADS = Hospital Anxiety and Depression Scale

CIS-20R = Checklist Individual Strength.

TOAST = Trial of Org 10172 in Acute Stroke Treatment.

Missing data in patients: education = 0.7%, NIHSS at admission = 1.4%

HADS-depressive symptoms = 0.7%, CIS-20R = 0.7%.

^a^Stroke could be located in more than one region in a patient.

^b^Group comparisons between the three groups of patients (left/right/infratentorial stroke).

**Fig 2:**
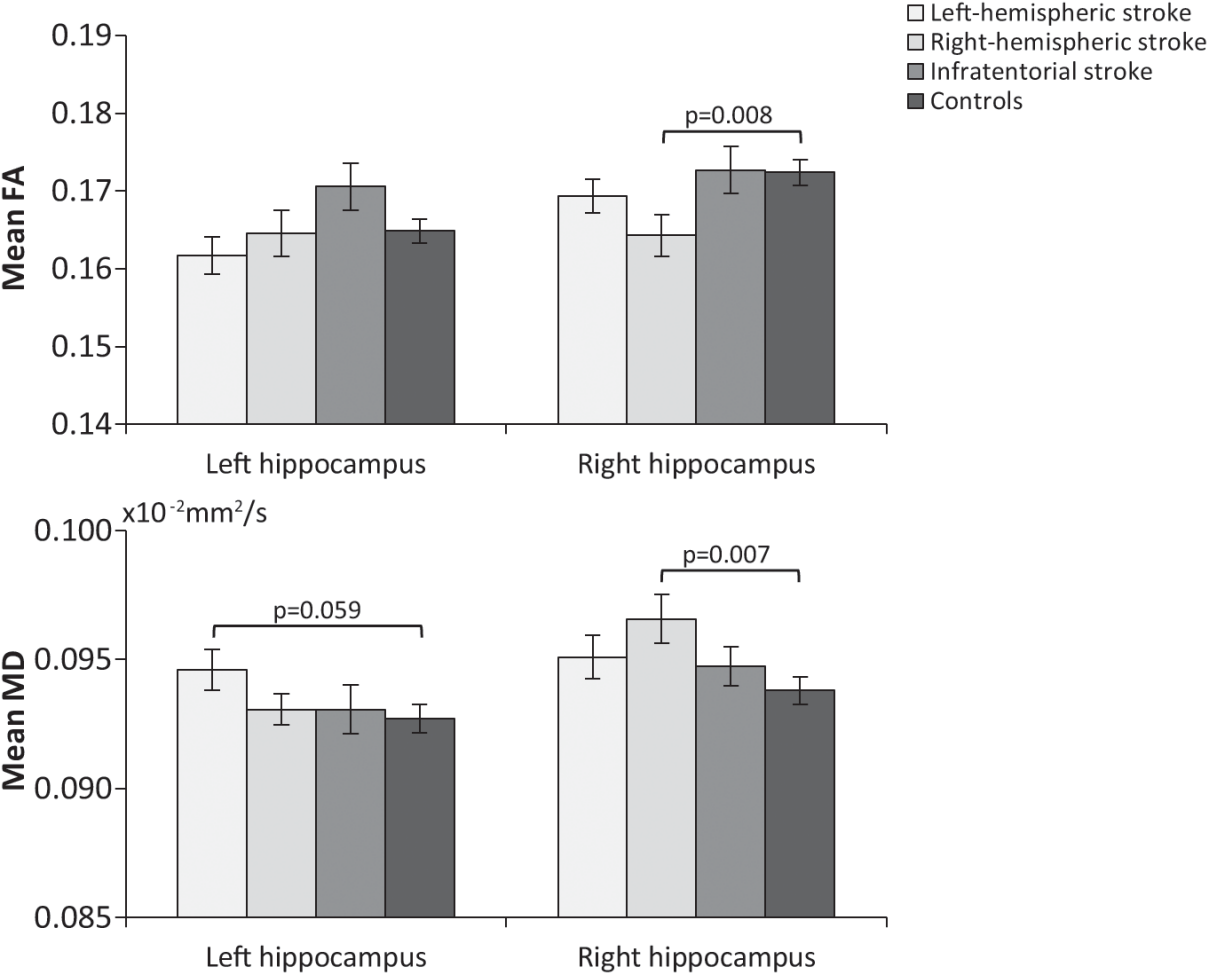
Unadjusted mean fractional anisotropy (FA) and mean diffusivity (MD) in the left and right hippocampus about 10 years after ischemic stroke in young adults compared with controls. p-values are adjusted for age at follow-up and sex.

We observed higher mean right hippocampal MD in right-hemispheric stroke patients compared with controls (p = 0.007), with a trend towards significance for higher left hippocampal MD in left-hemispheric stroke patients compared with controls (p = 0.059) (Fig 2). Right-hemispheric stroke patients showed lower mean right hippocampal FA compared with controls (p = 0.008). There were no differences in mean left and right hippocampal MD or FA between patients with infratentorial stroke and controls (Fig 2). We did not observe a relationship between ipsilateral hippocampal volume and ipsilateral hippocampal FA or MD in hemispheric stroke patients after adjusting for age, sex, follow-up duration, lesion location (left/right), and normalized lesion volume (p>0.05).

### Relationship between lesion volume and ipsilateral hippocampal integrity

After adjusting for age, sex, follow-up duration, lesion location (left/right), and ipsilateral hippocampal volume, larger hemispheric stroke volumes were associated with lower values of ipsilateral hippocampal FA (β = -0.38 p = 0.0002) and higher values of ipsilateral hippocampal MD (β = 0.26, p = 0.01).

### Patients with normal ipsilateral hippocampal volume and high versus low ipsilateral hippocampal MD

In left-hemispheric stroke patients with normal left hippocampal volume (less than or equal to 1.5 SD below the mean volume of controls) and significantly higher values of left hippocampal MD (F(1,36) = 38.28, p<0.0001), we did not observe a worse immediate verbal recall score (F(1,34) = 0.05, p = 0.8) or a worse delayed verbal recall score (F(1,33) = 0.35, p = 0.6) compared with patients with normal volume and lower hippocampal MD (Table 2). These two groups did not significantly differ in mean left hippocampal volume (p = 0.6) (Table 2).

**Table 2:** Comparisons between hemispheric stroke patients with normal ipsilateral hippocampal volume and high versus low ipsilateral hippocampal MD.

	Left-hemispheric stroke patients with normal left hippocampal volume			Right-hemispheric stroke patients with normal right hippocampal volume		
	Low hippocampal MD	High hippocampal MD	p-value^a^	Low hippocampal MD	High hippocampal MD	p-value^b^
No.	21	20		15	16	
Ipsilateral hippocampal MD (mm^2^/sec)	8.9x10^-4^ (0.1 x10^-4^)	9.9x10^-4^ (0.1 x10^-4^)	<0.0001	9.1x10^-4^(0.1 x10^-4^)	9.9x10^-4^ (0.1 x10^-4^)	<0.0001
Ipsilateral hippocampal volume (ml)	3.2 (0.1)	3.1 (0.1)	0.6	3.2 (0.1)	3.3 (0.1)	0.4
Immediate verbal recall (Z-score)	-0.6 (0.2)	-0.6 (0.2)	0.8	-0.3 (0.2)	-0.1 (0.2)	0.6
Delayed verbal recall (Z-score)	-0.4 (0.2)	-0.6 (0.2)	0.6	-0.1 (0.3)	-0.4 (0.3)	0.5

Data are presented as number or adjusted mean (±SEM).

^a^Comparison between left-hemispheric stroke patients with low left hippocampal MD versus high left hippocampal MD.

^b^Comparison between right-hemispheric stroke patients with low right hippocampal MD versus high right hippocampal MD. For the analyses on hippocampal volume we adjusted for age, sex, and follow-up duration. For the analyses on ipsilateral hippocampal MD we additionally adjusted for ipsilateral hippocampal volume. For the analyses on immediate and delayed verbal recall we adjusted for age, sex, education, follow-up duration, and ipsilateral hippocampal volume.

MD = Mean Diffusivity.

Right-hemispheric stroke patients with normal right hippocampal volume and significantly higher values of right hippocampal MD (F(1,25) = 27.07, p<0.0001) did not show a worse immediate verbal recall score (F(1,24) = 0.27, p = 0.6) or a worse delayed verbal recall score (F(1,24) = 0.56, p = 0.5) compared with patients with normal volume and lower hippocampal MD values (Table 2). These two groups did not significantly differ in mean right hippocampal volume (p = 0.4).

### Thalamic integrity after ischemic stroke in young adults

We used the thalamus as a control region to investigate whether the results were specific for hippocampal integrity or also apply to other ipsilateral structures.

After adjusting for age and sex, we observed a significant higher mean left thalamic MD after left-hemispheric stroke (0.087x10^-2^ mm^2^/s, SD 0.06x10^-3^) compared with controls (0.083x10^-2^ mm^2^/s, SD 0.05x10^-3^) (F(1,133) = 20.2, p<0.0001). We observed a higher mean right thalamic MD after right-hemispheric stroke (0.088x10^-2^ mm^2^/s, SD 0.06x10^-3^) compared with controls (0.084x10^-2^ mm^2^/s, SD 0.05x10^-3^, p<0.0001) (F(1,119) = 22.7, p<0.0001). No significant differences between hemispheric stroke patients and controls were found for the contralateral hippocampal FA and MD. Infratentorial stroke patients did not differ from controls in mean left and right thalamic MD or FA (p>0.05). In left-hemispheric stroke patients with normal left thalamic volume and significantly higher left thalamic MD ((F(1,30) = 41.69, p<0.0001) no difference in mean immediate recall score (p = 0.2) and delayed recall score (p = 0.6) was observed compared with patients with normal left thalamic volume and lower thalamic MD. These two groups did not significantly differ in mean left thalamic volume (p = 0.3).

In right-hemispheric stroke patients with normal right thalamic volume and significantly higher right thalamic MD ((F(1,15) = 14.87, p = 0.002) no difference was observed in mean immediate recall score (p = 0.4) and delayed recall score (p = 0.8) compared with patients with normal thalamic volume and lower right hippocampal MD. No difference between these two groups in mean right hippocampal volume was observed (p = 0.2).

## Discussion

We showed that after a mean follow-up of ten years, a first-ever hemispheric stroke at young age (18–50yrs) is associated with a lower ipsilateral hippocampal integrity compared with a non-stroke population. Larger lesion volume was associated with a lower ipsilateral hippocampal integrity independent of ipsilateral hippocampal volume. In a subset of hemispheric stroke patients with normal ipsilateral hippocampal volume and a significantly higher mean ipsilateral hippocampal MD no worse memory performance was found compared with patients normal hippocampal volume and lower hippocampal MD.

The strengths of our study include its large sample size and the long follow-up. We collected baseline and follow-up information according to identical procedures in all patients with high inter-rater and intra-rater agreement for hippocampal volumes. We used strict protocols for memory assessment and researchers were trained, in order to reduce the risk of information bias.

Some methodological issues of our study need to be considered [2]. Although the FUTURE study has a prospective design, the current analysis is cross-sectional and we therefore can only report on lower microstructural integrity after stroke compared with a non-stroke population. While this may be due to post-stroke microstructural degradation of the hippocampus, a longitudinal design is required to further support causality. However, our data demonstrate a relation between the stroke and a reduced ipsilateral hippocampal integrity, since we did not observe a lower microstructural integrity of the contralateral hippocampus. Furthermore, larger hemispheric stroke volume was associated with lower ipsilateral hippocampal integrity independent of ipsilateral hippocampal volume. Therefore, it is highly unlikely that these patients already had a lower microstructural integrity of the ipsilateral hippocampus before stroke onset.

Also, selection bias might have occurred, since patients who participated in the FUTURE study, but could not participate in de present DTI study had a poorer outcome compared with participants. However, selection would only have occurred when the relation between the stroke and microstructural integrity of the hippocampus would be selectively different in non-participants and this does seem unlikely [2].

The observed associations in the present study do not apply to all measures of microstructural integrity. The value of FA reflects the directionality of molecular displacement by diffusion and is influenced by crossing fibers [32]. These crossing fibers are present in the hippocampus [33] and thalamus [34]and influence the FA. Due to the intrahippocampal and thalamic fiber incoherence, low FA may not necessarily reflect underlying lower structural integrity [35]. Thus, crossing fibers in the hippocampus and thalamus might be an explanation for a lack of finding with FA but not with MD. MD reflects the magnitude of water diffusion which is less influenced by direction of fibers and therefore MD remains relatively constant and is more reliable for assessing microstructural integrity of brain structures with crossing fibers [3, 35, 36].

Another limitation of our study was that the DTI voxel size at MRI acquisition was relatively large. This could have caused cerebrospinal fluid partial volume effects in the small hippocampal and thalamic region of interest [35]. This is especially the case in patients with hippocampal or thalamic atrophy, which could induce higher MD values. We used eroded hippocampal and thalamic masks for our analyses and therefore it seems unlikely that partial volume effects had an influence on our results.

A limitation of the current approach is the cut-off of Z≤-1.5SD as “normal hippocampal volume” [30, 31] since older subjects with smaller volumes might still have normal volumes for their age [29]. However, we have tried to reduce this effect by dividing the controls into two equal groups based on the median age of the control group and subsequently determined “normal volume” per age category.

Our data clearly demonstrate a relationship between hemispheric stroke volume and remote effects on the microscopic level in the ipsilateral hippocampus. This relationship has also been found on macroscopic level of the ipsilateral hippocampus [2, 37]. Although the results on lower ipsilateral hippocampal integrity in left-hemispheric stroke patients were somewhat less convincing, showing a trend towards significance. This might be explained by our finding on the association between larger stroke volume and remote lower ipsilateral hippocampal integrity. As we observed larger stroke volumes in right-hemispheric stroke patients it might be that this resulted in larger reductions of ipsilateral hippocampal integrity in right-hemispheric stroke patients compared with left-hemispheric stroke patients. Interestingly, we did observe lower left thalamic integrity in left-hemispheric stroke patients compared with controls. Although thalamic stroke was excluded, this difference might be due to the location of the thalamus as it lies closer to the most common stroke location in our stroke cohort (MCA territory) compared with the hippocampus and therefore possibly disconnection due to stroke is more likely. Thus, our finding highlights that stroke volume is an important risk factor for remote effects on the ipsilateral hippocampus. A possible explanation for these remote effects may be the occurrence of spreading depression (SD) [37]. SD is the pervasive failure of brain ion homeostasis that transiently interrupts function of intact brain regions, which causes secondary neuronal damage and infarct expansion [37]. Another possibility could be disconnection of the hippocampus due to stroke in the connecting fiber tracts [38].

Another important finding of our study is that patients with preserved hippocampal volume and low hippocampal integrity at present do not show a worse memory performance compared with patients with normal volume and higher hippocampal integrity. Thus, the addition of low ipsilateral hippocampal integrity in stroke patients with normal hippocampal volume is not associated with a worse memory performance. Other studies in non-demented elderly did observe an association between lower hippocampal integrity and verbal memory performance, independent of hippocampal volume[3, 8]. In healthy elderly individuals a low hippocampal integrity before volume loss might reflect an early marker of underlying neurodegenerative disease, such as Alzheimer’s Disease [3, 8]. However, in young stroke patients the co-occurrence of Alzheimer pathology is very unlikely given their young age (about 10%) [39, 40]. Possibly, the variance in hippocampal MD values found in our young stroke population is smaller due to a different underlying mechanism and therefore no difference in memory performance was found. The course and extent of hippocampal damage might differ due to different underlying mechanisms and needs further investigation. Nevertheless, our results put forward the idea of a lower reserve in patients with a normal hippocampal volume on conventional MRI, but with lower microstructural integrity. They still might be at risk to develop MCI/dementia earlier, compared with a non-stroke population, especially when these patients come to an age where aging-related neurodegenerative pathology (amyloid pathology) occurs. Future longitudinal research should further investigate whether these young patients with higher hippocampal MD eventually develop MCI/dementia earlier compared with a non-stroke population [41].

Finally, our results suggest that hemispheric stroke has a generalized effect on ipsilateral brain structures remote from the infarction, such as a lower ipsilateral hippocampal and thalamic integrity. This is further confirmed by previous studies on hippocampal and thalamic volume after ischemic stroke [2, 24, 25, 37]. Lower microstructural integrity in remote structures has also been found in subjects with cerebral small vessel disease [42–44]. Thus, these results in subjects with cerebral small vessel disease and stroke patients provide evidence that beyond the area of infarction, remote effects of subcortical damage occur.

## Conclusions

In conclusion, our data suggest that vascular lesions are associated with remote lower ipsilateral hippocampal integrity. At present, patients with normal volume and lower hippocampal integrity do not show a worse memory performance compared with patients with normal volume and higher hippocampal integrity. Possibly, a history of stroke, combined with brain changes associated with normal ageing, might eventually lead to exacerbated memory decline. Our findings add to the increasing awareness of stroke not only being an acute disease with immediate consequences, but also results in life-long, consequences.
